# The Physician King Buddhadasa: A Mine of Virtues, as the Sea is of Jewels

**DOI:** 10.7759/cureus.71416

**Published:** 2024-10-14

**Authors:** Shehan Silva

**Affiliations:** 1 Department of Medicine, Faculty of Medical Sciences, Colombo South Teaching Hospital Kalubowila, University of Sri Jayewardenepura, Colombo, LKA

**Keywords:** historical vignette, humane medical practice, medical ethics, medical professionalism, sri lanka

## Abstract

King Buddhadasa was a monarch of the Kingdom of Anuradhapura, Sri Lanka, in the 4th century A.D. He was a unique king with sound knowledge and skill in medicine according to the traditional medical system. The primary source of Sri Lankan history describes him as "a mine of virtues, as the sea is of jewels." This is due to his virtue of being a righteous king and a compassionate physician, whose healing extended to both humans and animals. His contributions went beyond curative medicine to include preventative care, medical administration, and scholarly work such as the Sarartha Sangrahaya. Similar to Charaka and Sushruta in the South Asian region, his life and work are exemplary and inspirational to modern-day physicians. This review provides an account of the life of King Buddhadasa as recorded in the primary chronicle of Sri Lankan history. Some of his healing accounts may seem mystical to the reader. The article also endeavours to find plausible explanations for those occurrences based on allopathy and science. These records serve as important lessons for modern-day physicians, encouraging the inculcation of good conscience and morality in medical practice.

## Introduction and background

A unique system of medicine existed in Sri Lanka, known as Desiya Chikitsava or Paramparika Vedakama (meaning traditional or native treatment in Sinhala), before the advent of Ayurveda. This system, still practised on the island, is also referred to as "Sinhala treatment" (Hela Vedakama or Sinhala Vedakama), reflecting its ethnological roots parallel to the evolution of a community-based healing system. The system was based on the knowledge and experience of its practitioners and was handed down from generation to generation in the form of master-apprentice (Sinhala: guru-shishya) instruction. There is evidence of the existence of hospitals in places such as Mihintale and Medirigiriya based on this system. The use of medicinal troughs and surgical equipment, comparable to those used today, is strong evidence of this. The system later integrated with Ayurveda to such an extent that the Ayurveda Act of 1961 in Sri Lanka acknowledged the existence of traditional or native treatment. A few practitioners, termed vedaralas, especially in rural Sri Lanka, kept it within their families with some secrecy (Sinhala: guru mushti).

Physicians in ancient Sri Lanka were highly respected [1]. A proverb in Sinhalese society states, "If one cannot be a king, be a physician." The profession of healing was paralleled to that of royalty. Some noteworthy kings practised medicine to dignify their rule, adding prominence and uniqueness to their conduct. Among them, Ravana from prehistoric times and Aggabodhi VII and Parakramabahu I from recorded history, play prominent roles in the island's history. King Buddhadasa is another important name in the hall of fame of physician kings.

The reign of kings is primarily recorded in the Mahavamsa (Greater Chronicle), which is composed of the Mahavamsa proper and the Culavamsa (Lesser Chronicle). These are the widely accepted primary historical sources of the nation. Additionally, the chronicles contain liberal evidence depicting medical facts that can be linked to the traditional medical system of Sri Lanka. Valuable information can be gleaned from these pages, albeit with elements of exaggeration in the form of fantasy, mysticism, and metaphysics, influenced by native and Buddhist cultural practices. The record of King Buddhadasa in the Culavamsa contains many such strange yet intriguing stories [2,3]. However, these accounts need to be taken at face value when understanding the possible pathophysiology linking to scientific medicine, as discussed below. This review delves into some of these accounts, providing plausible explanations linked to modern medicine.

The personalities mentioned in the chronicles are described through the lens of religious virtuosity and piety, as they were written by Buddhist monks. Sovereigns who reigned with righteousness, providing care for their subjects in terms of health, nutrition, and welfare, are depicted with high levels of praise and honour. Buddhadasa, too, was greatly praised for his life as a ruler and his service. The authors of the Culavamsa placed him among the greatest kings, saints, or monks who shaped the history of Lanka, dedicating no fewer than 74 stanzas to him, even though he was neither a warrior nor a builder of temples or lakes. Such qualities are indeed important lessons for all those who practise medicine today. This review considers these qualities as valuable lessons for modern-day medical professionals to become better physicians and human beings. Buddhadasa's fame for medical prowess is known both in Sri Lanka and beyond [4].

## Review

The king who was a physician

King Buddhadasa was a monarch of the Kingdom of Anuradhapura (an ancient Sri Lankan capital, now a sacred city) from the year 341 to 370 A.D. He ascended to the throne through lineage from the Lambakanna dynasty after the death of his father Jetta Thissa I. The king was gifted with exceptional knowledge and skills in medicine [3]. There is no account of the source or even the name of a teacher from whom he acquired this. However, it can be thought that he may have been mentored by a well-known physician in the master-apprentice tradition.

The Culavamsa provides a generous description of Buddhadasa. He was equalled to Kubera (Vaisravana), the god of wealth as he ensured happiness, prosperity and security to all his subjects. He showered mercy upon all as a father who has compassion for his children. Buddhadasa was gifted with both wisdom and good virtue. He was considerate of the human and social aspects of healing which are pivotal to physical relief [5]. This may be the reason why the author of Culavamsa described him as "a mine of virtues as the sea is such of jewels". 

The virtues of the physician king

The chronicle states that he ruled with dasa rajadhamma: the tenfold virtues of rule. These, as stated in the Khuddakanikāya sutta, are charity (dāna), morality (sīla), altruism (pariccāga), honesty (ājjava), gentleness (mandala), self-control (tapa), non-vengeance (akkodha), non-violence (ahimsa), forbearance (khanti), and uprightness (avirodhana). Such virtues were considered to be a tower of refuge of supreme piety [6]. The tenfold royal attributes are characteristics that a modern-day physician should consider fostering and demonstrating in his life and profession. Medical professionalism is based on the concepts of morality, ethics, and good faith. The American Board of Internal Medicine, along with the American College of Physicians, released a Physician Charter in 2002 [7]. This was based on the principles of patient welfare, patient autonomy, and social justice. The qualities of dasa rajadhamma that King Buddhadasa upheld intersect with the 10-fold principles of medical professionalism in the Physician Charter.

Buddhadasa was free of the four unprofitable directions (satara agati). These are volition (chanda agati), hate (dosa agati), fear (bhaya agati), and delusion (moha agati). He was praised for winning the hearts of those he encountered through charity (dāna), kindly speech (peyyavajja), beneficial conduct (attachariya), and equality (samanattata): the chattari samgahavattuni [6]. Buddhadasa, being a Buddhist, would also have upheld the sacred tenets of its foundations. Compassion (karunā) with equanimity (upekkhā) is important to pave the way for one to understand and relieve or attempt to relieve the pain and distress of others. It springs to life while maintaining calmness to prevent burnout and improve patient care [8]. Mindfulness (sati), also taught in the same philosophy, calls professionals to be present in themselves and be attentive, facilitating empathetic and effective doctor-patient interactions [9]. Integration of trust, compassion, and justice from the philosophy of Buddhism into the practice of medicine is relevant [10]. Such attributes are important virtues needing edification in the life of anyone availing himself of the profession of medicine. Thus, the king's life was exemplary for modelling professional behaviour, and more so for being a humane doctor.

The great king had an unconcealed life, so people had faith and trusted in him. Thus, he maintained the duty of candour to both his subjects and patients. His selflessness was shown by his benevolence towards the vulnerable and deprived, with a duty to protect their interests. Buddhadasa was also an amicable person who treated the deserving with cordiality; however, the wicked were dealt with sternness.

Healing of a King Cobra

**Figure 1:**
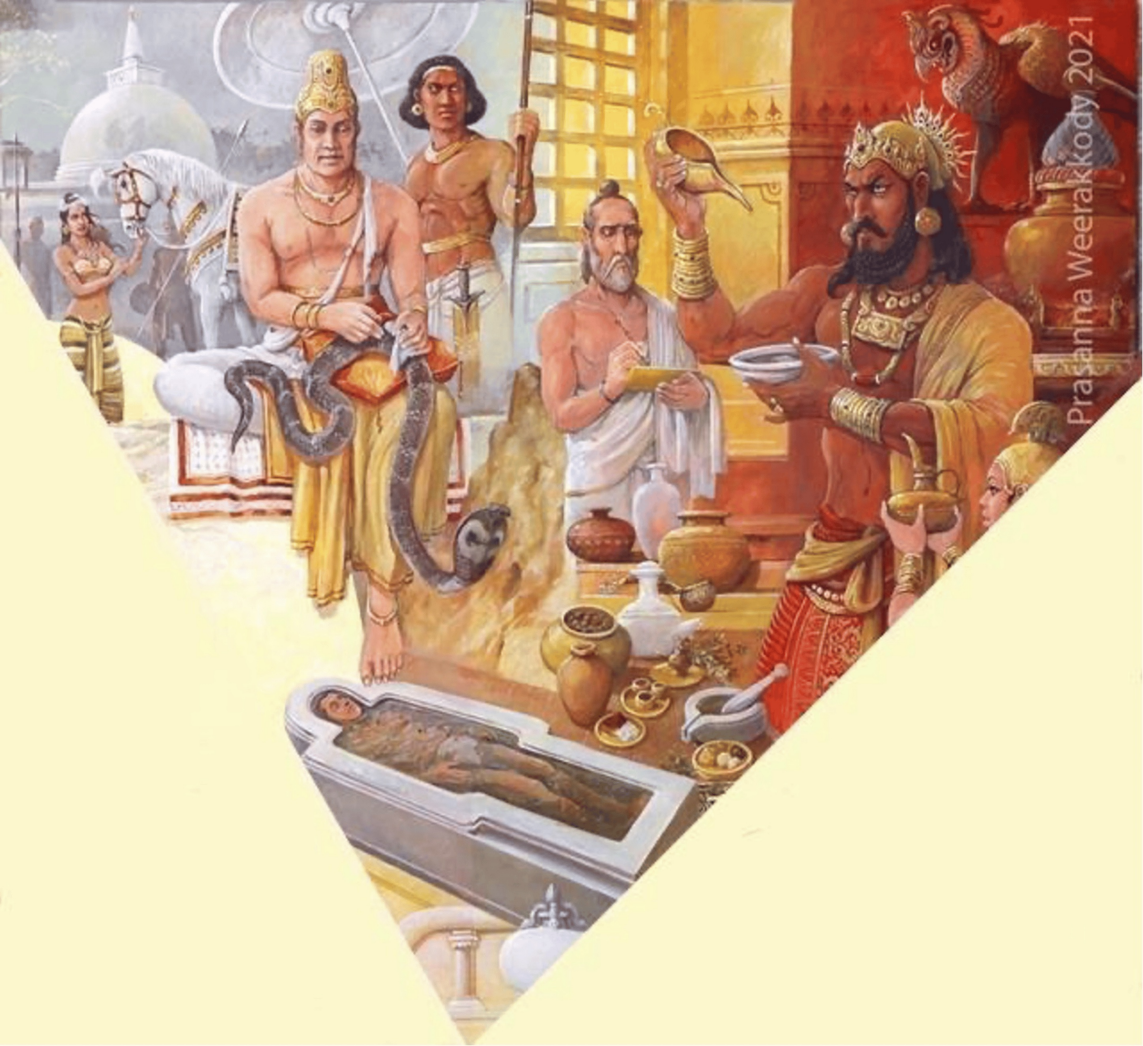
A section of a painting titled "Ayurveda/Hela Veda Roots of Lanka". Obtained with kind permission from the artist Prasanna Weerakkody (2021). On the left side, King Buddhadasa is depicted treating the king cobra while on the right King Parakramabahu I, another physician king, is shown preparing medication.

Compassion and empathy were part of the monarch’s very nature. Buddhadasa’s encounter with a king cobra is perhaps the greatest story ever written about his fame (Figure 1). One day, on his way from the Royal Bath at Tissa Wewa Lake, he saw a king cobra (Naja naja) afflicted with an infirmity of the abdomen. The animal was lying with its belly upward due to a painful tumour. Buddhadasa dismounted his elephant and had a discourse with the serpent in clinical and logical reasoning. He expressed that he understood the torment, but also stated that he was hesitant to relieve it as snakes are easily enraged and may retaliate. The king was not able to alleviate the suffering due to the inevitable iatrogenic pain that would result from using a scalpel. Buddhadasa was mindful of the harm his treatment might cause despite the potential benefit to the animal. The king cobra understood his reasoning and inserted its head and hood into an anthill to remain motionless. The physician king took out his side knife, similar to a scalpel (satthavattim in Old Sinhala), which he always carried, and excised the tumour. He then applied medicament to relieve the pain. Snakes are believed to possess precious jewels (nagamanikya in Sinhala), which they protect with reverence as if guarding their lives. These jewels were used in ancient medicine to extract venom [11]. Modern medicine discourages this practice, but the folklore surrounding these naturally occurring animal bones is still considered metaphysical and miraculous [12,13]. The Culavamsa mentions that the snake, in gratitude, presented the king with its jewel. As per tradition, the king later placed the jewel upon a stone image of Buddha at the Abhayagiri temple. Buddhadasa himself acknowledged that the beasts understood his charity and good intent due to his right conduct in ruling [6].

Some accounts of healing

A Buddhist monk consumed some milk that was presented as alms during a mendicant round. The meal, which was contaminated with worms, made him sick. He sought refuge from the king, who discerned the problem through clinical reasoning (roga nidā​na in Sinhala). At the time, the king was treating a horse by bloodletting. The monk was made to ingest the products of the venesection. Shortly afterwards, he was informed of the origin of the medication, which induced immediate emesis due to disgust. The pathogen was evacuated, and the patient was cured. This account demonstrates the monarch's knowledge of the principle of inducing emesis, which is practised on those intoxicated. The king successfully treated both man and beast with a single stroke of a knife [6]. On another occasion, a monk afflicted with a disease that caused writhing, cramps, spasms, or contractures, resulting in a shape resembling a roof, was also treated by the king.

A man was afflicted with a disease after ingesting a water snake egg. The chronicle states that the hatchling attached its mouth and sucked the life from within the man. Unaware of the cause, the man consulted the king’s physician, who diagnosed the condition aptly. The patient underwent fasting, medicinal bathing, and massages. Once adequately sedated, a string with a piece of meat was lured into the gastrointestinal tract by the king. The snakeling bit into the meat, and the king successfully retrieved the animal [6]. Long roundworms and tapeworms had a significant impact on public health in the ancient world. A well-known method of treatment, still practised today, involves extracting the long helminth intact from the patient [14]. The Guinea worm nematode, Dracunculiasis medinensis, is postulated to be the fiery serpent mentioned in the Bible [15]. The "snake" described in the Culavamsa in this story could be considered the same, or it may refer to Ascaris lumbricoides (roundworm) or another long platyhelminth. In the end, the author of the Culavamsa states that the king's practice of medicine was on par with that of Jivaka, the physician of Buddha, performing it with loving-kindness (mettā).

Buddhadasa also had a sound understanding of midwifery and obstetrics. A woman of the untouchable Chandala caste was pregnant for the 8th time, and the pregnancy was malpresented. The king intervened [6]. Society had a taboo against associating with those of lower castes, but the king stooped down to treat this woman, who may have been a beggar, a sweeper, or a scavenger. This account highlights the non-discriminatory and non-bigoted behaviour of the physician-king, who treated anyone in need of his service.

A man consumed water contaminated with "frog eggs" and was later tormented during the monsoon season with headaches caused by frogs that had allegedly entered his skull. Buddhadasa performed a craniotomy to release the animals and then closed the incision, approximating the bones [6]. A plausible explanation could be parasitic infections such as cerebral echinococcosis, neurocysticercosis, or cerebral toxoplasmosis, misunderstood by the author of the Culavamsa [16]. Whatever the pathogen, the king had the knowledge and skills to perform neurosurgery to relieve the man of his disease.

There was a leper who was an enemy of the physician-king. Buddhadasa attempted to reconcile with him. Lepers were shunned in ancient society as untouchables [17]. The king directed one of his men to befriend the leper and share in his resentment. The king, pretending to be against himself, invited the leper to stay at his house, claiming he would destroy the king with the leper’s help. The leper was bathed, well-fed, and taken care of. As he became happy, contented, and calm, the attendant offered food and drink, stating that it was a gift from the king. The leper rejected the medication twice, but on the third occasion, he accepted it and was directed towards complete healing. Thus, Buddhadasa reached out not only to the physically diseased but also to a person mentally afflicted with resentment against him. Healing of the mind and upliftment of spirituality were achieved due to this ingenious method. This account clearly demonstrates compassion, empathy, and the opportunistic approach that a physician should adopt to heal any human being without prejudice or bias. The king had feelings for the patient and shared in those feelings with him [6].

Sarartha sangrahaya: a medical treatise

**Figure 2:**
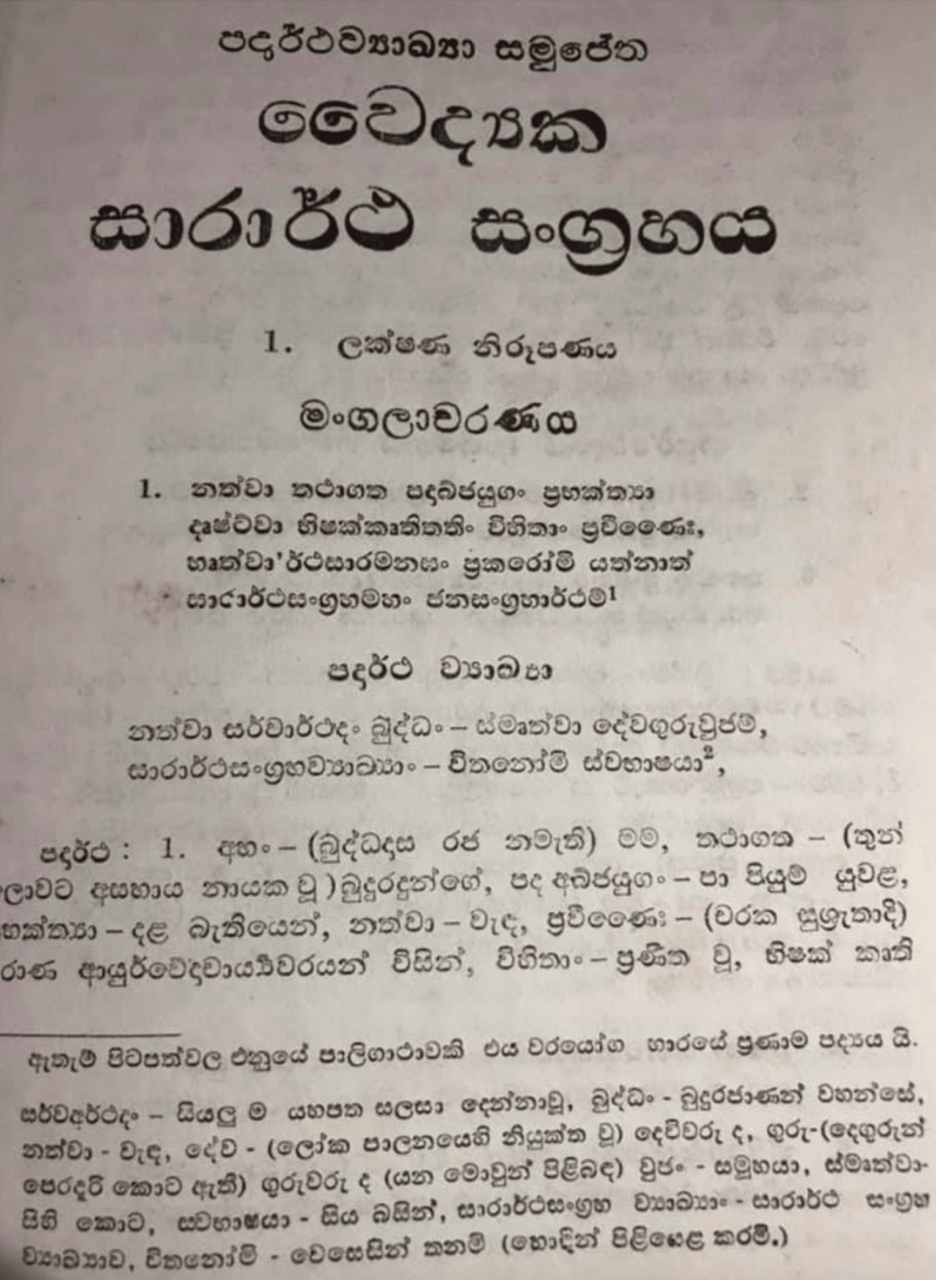
The preamble of the Sarartha Sangrahaya. The first stanza states, "I compile the Sarartha Sangrahaya in my mother tongue after venerating the Buddha and remembering gods, parents, teachers and my subjects". Photographed by the author.

Buddhadasa was an erudite scholar. He compiled a compendium in Sanskrit named Sarartha Sangrahaya, which contained instructions on clinical reasoning, therapeutics, and pharmacopoeia (Figure 2) [3,6,18]. Diseases that were given prominent attention included tuberculosis, snake bites, skin diseases such as psoriasis and leprosy, and even psychiatric disorders. The book also describes the technology of that time, including the surgical equipment used to treat patients. This comprehensive work, along with Bessajja Manjusawa (a 13th-century work by monk Chanawimla), is still used in desiya chikitsava in Sri Lanka. Sarartha Sangrahaya is considered similar to Susrutha Samhita in its organisation and comprehensive compilation [19,20].

Beyond curative medicine

Buddhadasa had the wisdom to identify ways to improve public health and sanitation to control the spread of disease. He appreciated the importance of the concept that prevention is better than cure. In an administrative capacity, he appointed a physician to care for 10 villages and set up "places of refuge," similar to modern-day clinics or hospitals. These institutions were organised and provided with state patronage and grants [4]. There were separate places of refuge for those who were challenged, including the disabled and blind [3]. He also looked after the well-being of his armies, adhering to the principles of military medicine [21]. Furthermore, the king, understanding the importance of holistic care and spiritual health, appointed lay preachers to provide healing for the mind and spirit. The royal physician also extended care to veterinary health, even for animals such as horses and tusker elephants, following the concept of "One Health" [21]. The salaries of physicians were systematically ordained as well [3,6]. The king is also credited with establishing reserves as botanical parks to grow medicinal plants. The Nilgala forest reserve in Monaragala district is one such park that still exists today.

## Conclusions

The name Buddhadasa has a significant meaning "the servant of the Buddha". Gautama Buddha once discoursed, "If someone cares for a sick person, he is the one who cares for me". True to these words, Buddhadasa acted with altruism, humility, and dedication in bringing healing and comfort to both beast and man. Physicians, as well as all Sri Lankans, are well aware of this monarch's legacy and hold a high level of respect and adoration for his memory. King Buddhadasa is indeed an important figure in South Asian medical history, akin to Susrutha and Charaka of the Ayurveda tradition. His humane qualities are valuable lessons that physicians, at all times, should consider exemplary in shaping their professionalism and practice.
